# Elderly Men’s Experience of Information Material about Melanoma—A Qualitative Study

**DOI:** 10.3390/healthcare1010005

**Published:** 2013-07-11

**Authors:** Kristina Rosengren

**Keywords:** malignant melanoma, men’s experiences, person-centered care, qualitative methods, self-care learning, skin self-examination

## Abstract

Malignant melanoma is an aggressive disease that has been increasing worldwide. Public education is trying to focus on reducing intense sun exposure and raise awareness of signs and symptoms to prevent illness. The aim of the study was to describe and analyze elderly men’s (over 65 years) experience of an information booklet regarding malignant melanoma. The study comprised of a total of 15 interviews with elderly men. The interviews were analyzed using manifest qualitative content analysis. Respect for the individuals was a main concern in the study. One category, *Security—to act*, and three subcategories, *Availability—to use*, *Clarity—to understand*, and *Awareness—to know*, were identified to describe the men’s experiences of information material about melanoma. By using person-centered care, based on a holistic approach focusing on men’s need for security to act on specific risk factors and to do skin self-examination, health could be improved. The results of this study could help other health organizations to develop information material to prevent illness, such as for skin self-examination. Strategies concerning educating, preparing, and training health professionals in interpersonal communication skills should be implemented in healthcare organizations to meet patients’ information needs about illness to develop continuous learning and quality improvement.

## 1. Introduction

Malignant melanoma, an aggressive disease that is increasing worldwide, is one of the top ten identified diseases [1]. People with fair skin are a risk group due to the sun’s ultraviolet rays that can cause damage to skin cells; another risk factor is having a family history of melanoma [2,3,4,5]. Self-reported information about skin cancer among elderly people could be the only available source of health data [6], thus indicating the importance of educating people about risk factors and how to perform skin self-examinations. Hodgetts [7] also stresses that public education, focusing on reducing intense sun exposure and skin examination, could raise awareness of signs and symptoms of an early onset of the disease. Education needs to be both practical and specific due to patients’ needs and the diagnosis of malignant melanoma [8]. However, a study from 2004 showed that awareness of sun exposure and development of malignant melanoma do not reduce melanoma, though they may increase the use of sun protection [9]. Moreover, it is important to diagnose and treat malignant melanoma at an early stage. Therefore, early diagnosis and treatment to identify high-risk patients is recommended as a secondary prevention; this could also be described as cost-effective for the society [2,3,4,10]. Treatment at the onset of the disease is surgical, and the patient therefore needs to be informed about the risk of complications in accordance with the right to expect performance of best practice [11].

A study of health promotion in New Zealand showed that males have specific needs but they do not always seek help to monitor their health issues. Building a healthy public policy, creating supportive environments, strengthening community action, developing personal skills, and finally re-orientating health services models, built on the Ottawa Charter, resulted in effective health promotion and effective use of limited resources [12]. A study of elderly men with prostate cancer showed that they do not ask for information about their health and ill health, which could be seen as lack of trust in the practitioner or dissatisfaction with received care. Another point of view could be that the masculine characteristics make them reluctant to ask for help or advice [13]. However, another study of men with prostate cancer showed that they experienced and responded emotionally, but a deeper understanding of sense of self and ideas of manhood were implied [14]. In another study, outpatients infected with HIV/AIDS were described as having learning preferences with a broad and holistic view related to prevention and self-care learning needs that empowered them to be active co-planners in care [15]. Men often visit a physician due to their partner’s anxiety [16]. Moreover, a literature review pointed out that psychological stress, as well as coping strategies, were important factors to consider in ongoing nursing [8]. To create best possible health outcomes, an interaction between health professionals and patients (HIV/AIDS) is needed, where the motivation, knowledge gaps and specific health needs of the patients enhance behavioral changes, as well as skills where the health professionals teach them self-management strategies [17]. There is a lack of literature regarding information needs of men with malignant melanoma and the effects of care delays and quality of care. In Sweden, approximately 2,300 people each year develop malignant melanoma and the incidences for elderly men (over 65) are three to four times higher than in the general population. However, there is no clear answer to these gender differences. The median age is 60 years for females and 64 for males. Melanoma has increased during the past 10 years (4%) compared with non-significant changes in the 1990s. The mortality rate has also increased from 4/100,000 in year 1999 to 5.2/100,000 in year 2009, from 348 to 499 deaths. In general, men from Sweden and Denmark have high mortality rates. However, increased awareness of how to detect melanoma and easy access to treatment may allow patients with thin melanomas to attain almost 100% survival [18,19].

A study among female patients stresses the discrepancy between what health professionals view as anticipated educational needs for patients and patient’s needs. Coping skills and nutrition were ranked higher by the patient than by the physician [20]. Nurses are described as efficient information providers compared to other healthcare professionals. Information in regards to diagnosis and prognosis treatment, as well as care, needs to be well planned and delivered with emotional support before consultation, surgery, and education. A nurse giving advice and education will provide emotional support in relation to the patient’s faith and hope [8,10]. Moreover, nurses should be specifically educated, prepared, and trained in interpersonal communication skills to be able to meet patients’ information needs [21]. The degree of social support was explained by the differences in the caregiver burden, level of education and stress. Furthermore, a high degree of a caregiver’s education and cooperation with the primary care clinic was related to a low caregiver’s burden, and low education was related to a high caregiver’s burden. Social support was a main factor in perceived caregiver burden [22]. Information and support to older patients with cancer, family members and friends require optimal assessment of needs and referral to appropriate services [23], that is why it is significant to administrate and guide healthcare staff the best way by being present and available in the daily work [24,25].

Since men are less likely to seek help and care for their health situation, it is important for health professionals to pay particular attention to men in order to improve their quality of life, taking into consideration both public health and economical cost effectiveness [1]. Another point of view is the gap in the management of chronic disease in older patients according to elderly patients’ needs, situation and preference [26]. However, there is a lack of studies about men’s experiences of melanoma, why it’s interesting to improve knowledge in the area to use limited resources in healthcare in the best possible way to decrease the mortality rate among elderly men. Health outcomes in the elderly population is a complex phenomenon affected by social organization and life habits, as well as economic development [27]. It is therefore interesting and valuable to educate elderly men to prevent ill health. The aim of the study was to describe and analyze elderly men’s (over 65 years) experience of an information booklet about malignant melanoma.

## 2. Experimental Section

### 2.1. Design

This study was a qualitative study to determine older men’s understanding of melanoma in terms of how to recognize it, self-examine,* etc.* through use of a new information booklet. Qualitative research requires insightful and artful interpretation and is dependent upon trustworthiness, transparency, verification, reflexivity, in addition to being necessarily participant-driven [28].

### 2.2. Setting

The study was set up to evaluate a project to prevent or diagnose early malignant melanoma. The cancer incidence rate for malignant melanoma in the year 2011 was 5.5% (Sweden), which included a total of 1,676 males, 83 of whom lived in the county (129,688 people) in the southern part of Sweden. The study was conducted in one municipality within a county with 17,366 people, an urban area whereby 13% of the population is over 75 years. An information booklet was sent out by mail to all men. There were 306 persons over 65 years who were associated with the two primary health centers in the municipality. The information booklet was newly developed about skin cancer and produced to educate men over 65 years about malignant melanoma. The information material content included details about skin changes in shape and color, contact information of health clinics, and referrals for further information. 

### 2.3. Data Collection

Men (306 persons), were invited to participate by mail. The first 15 men that reported interest in participating either by mail or by telephone were included in the study, regardless of current diseases or cancer diagnoses, including melanoma. The author contacted the participants by telephone, and the data collection took place from May to June 2012. All interviews were carried out in privacy in a room according to the participants’ wishes, in, for example, their own home or in a public place, and were conducted by the author. The questions were based on a thematic guide, with specific fields of questions concerning the men’s experiences of distribution methods, amount and content of information, images, layout, quality improvement and future information needs. The study comprised of a total of 15 semi-structured interviews (Table 1) and lasted between 20 and 60 min, and were tape recorded and transcribed *verbatim*. No background information regarding earlier diseases was collected.

**Table 1:** Description of the study group with regard to age and marital status.

**Age**	**Numbers**
65	6
70	6
75	3
	*n =* 15
**Marital status**	**Numbers**
Married	11
Cohabiting	1
Single	3
	*n =* 15

The interviews began with background questions and ended with a final question to summarize the participant’s experiences. Each theme started with a question such as: “Could you tell me about X?” Further questions were based on the participants’ answers; they were asked about their experiences of a newly developed information booklet, as well as their information needs in relation to malignant melanoma. Examples of situations, clarifications, and further elaborations were requested. The data collection focused on the elderly men’s experience of information about malignant melanoma.

### 2.4. Data Analysis

The interviews were analyzed using manifest qualitative content analysis, suggested by Egberg *et al*. [29], as a step-by-step procedure. Written words were used (from the interviews) as the basis for the analysis. Texts were read to acquire a first impression of the content. The manifest analysis addressed questions about experiences from information material about malignant melanoma. The analysis (Table 2) was performed in the following steps: (1) Transcripts were read and re-read to obtain an understanding of and familiarity with the text; (2) Meaning units (words, sentences or paragraphs) corresponding to the content areas were selected for (a) information structure, and (b) security; (3) Each meaning unit was condensed into a description of its content and labeled with a code; (4) Subcategories were identified and grouped into categories, *Availability—to use*, *Clarity—to understand*, and *Awareness—to know*; (5) One category, *Security—to act* formed the main area.

**Table 2:** Example of description of analysis of content into subcategories that formed a category.

Meaning unit	Condensed content	Coding	Subcategory	Category
... I thought it was positive that caregivers act within the area, that was my thought. I could see my body but I have not found anything ... but I’m not exactly worried ... but as I said, I think it’s positive ...	Positive that healthcare is engaged in educating people	Knowing what skin cancer is	Availability—to use	Security—to act

The emerging findings are illustrated through the participants’ quotes.

### 2.5. Ethical Considerations

An ethical approval and permission for the study was obtained from the managers of the municipality and also from the regional ethical review board. Respect for the individuals was a main concern during the study. All informants were informed about voluntary participation and consented to participate in the study, knowing their right to withdraw at any time, and that their answers would be kept confidential. Respect for the participants’ integrity and autonomy was therefore shown. Ethical guidelines for human and social research have been followed throughout the study [30].

## 3. Results and Discussion

### 3.1. Results

**Figure 1:**
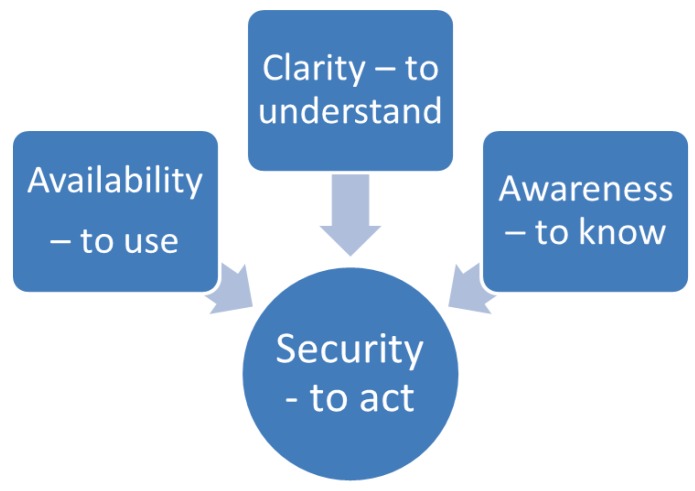
Show the results four categories.

The result consists of four categories: “Availability—to use,” “Clarity—to understand,” “Awareness—to know,” and “Security—to act.” This is presented by Figure 1.

#### 3.1.1. Security—To Act

The main category, “Security–to act”, describes elderly men’s need of knowledge in the field of malignant melanoma, which contributes to security related to variation among skin changes due to age. Security to act was described by the informants as a tool to prevent illness related to a variety of diseases that elderly men may suffer from. Men described themselves as not easily frightened, referring to the fact that the their life is mostly behind them; this is also described as male vanity that can hide fear of illness and future health interventions. Moreover, elderly men describe different experiences of cancer and an interest to know more in order to provide security. Another aspect that contributes to security was their relationship with caregivers that are of a predominantly positive nature. Participants point out the importance of receiving feedback from caregivers to feel secure to prevent illness. 

Cancer is a loaded word that can give a sense of fear and anxiety; however, the information booklet has contributed to the participants’ security regarding awareness and preparedness. Men emphasize that the information booklet was clear since it consisted of limited body text with color images, which improved readability and understanding of the content in relation to their own skin. The informants stressed that the information booklet has become a tool that gives them a sense of security in terms of self examination of skin status. They can know, use, understand and act on changes in their skin in a different way than before. Moreover, information about how to do skin self-examination and to act on suspicion made them secure as illustrated by the following quote:

*“ ... I thought it was positive that it takes care of that problem, that was my thought, but I see the greater part of my body and I have not found anything ... but I’m not exactly worried due to no particular or way to do it I heard (laughs) ... no, I am not worried if I get home like this but as I said, I think it’s positive ...”*

Another important aspect was that the information booklet was sent home to participants through a personal letter from the County Council so they could save it, thereby contributing to security when the information was preserved in the home environment. 

#### 3.1.2. Availability—To Use

The category “Availability—to use,” describes a participant’s need to use information in everyday life. Elderly men describe that the distribution methods by regular mail addressed with their names in an envelope from the County Council was an excellent way to reach the majority of men over 65 years. Participants also highlighted the need for repeating information, for example, postcards, to keep the interest alive and to reach all men. All men (65 years old) should be included so they could use information in the best way. The participants highlighted the importance of information dissemination; one aspect that they describe is the County Council’s resources, which is why they argue for sending postcards, since they have a positive value and could be a cheaper alternative to letters. However, informants described a skepticism of the use of media in order to increase the availability of information, and also the importance of not confusing this information with mailshots. This is described by the quote below:

*“... It is important to send it right to me (a target group of men over 65 years) and there’s no living man who does not open a letter, for me it’s quite obvious, if it does not perhaps show that it is advertising for any crap that I’m not interested in, then I throw it away ... Can you afford it and if you have the ability and technology to address it to the audience you want to reach, then it’s best ...”*

Computer-based mailing has its limitations until computer access has increased within the target group. Another aspect that participants stress is how easy it is to sort out electronic mail in relation to the paper material that requires more work. Other distribution methods to improve accessibility for information that also have limitations are libraries, sport events, pharmacies, health centers, and the waiting rooms of hospitals.

Moreover, the participants described a need for information focusing on the male population. They described delight to be recognized as men over 65 years, but they also raised the question of why “only” men are included compared to women who often sunbathe more than men. Another aspect that informants raised is male self-consciousness, that is, that men pull away and do not want to be noticed. Informants reported that they have had some experience of cancer themselves, or a family member and friend, which has influenced them to seek the benefits of using the information in the best possible way. However, participants highlighted that information is important since cancer is a negatively charged word. The information material is described as simple and effectively structured because of a three-part A4 page giving six separate “pages,” a handy format that improves accessibility to the information. Informants also pointed out the possibility of performing skin self-examinations in their home environment due to the available information which they could read in peace and quiet repeatedly, and compare with the pictures in the brochure with their own skin.

#### 3.1.3. Clarity—To Understand

Category “Clarity to understand,” described the participant’s need of readable information material that describes basic knowledge of skin cancer. The elderly men highlighted the exemplary short text, which is followed by a number of colored pictures that they could connect to when examining their own skin. They described that the text was easy to read, focused on malignant melanoma and went straight on without any restraining body text. Informants stressed the objectivity in both text and images with a serious purpose, which allowed the reader to become aware of the illness. They also described the importance of natural images of different skin changes to understand and compare to their own skin and its changes. Informants pointed out that those pictures easily remained while the text was lost from memory; an information booklet that is full of images will contribute to clarity of understanding. One participant puts it this way:

*“... You embrace so much more from an image than from a little text, a text you just skim through and you might not read anything special but a picture catches you a little bit differently, so I think this is really good and also you can see then if there is anything that you yourself may have to do ...”*

The introduction “Information about skin cancer” is not perceived by the informants as frightening or disturbing. Rather, it helped them to understand why they received the information material. Another good aspect was that the booklet did not point out separate things, which otherwise might have adverse effects that do not contribute to clarity of understanding. Men stressed that their understanding of various forms of age-related changes in the skin had increased, such as age warts. Further improvements could be to develop the body text related to the different pictures, by, for example emphasizing changes, itchy red skin, freckles and the sun’s harmful effects. However, informants emphasized the importance of short passages of body text. Moreover, the men pointed out that society should be better at discouraging sun tanning, but they also think that it is difficult to avoid the sun. Participants described that clarity could be increased if the information about the content about melanoma were to already appear on the envelope. The content could increase interest and understanding and could motivate one to take care of the information material. Another point of view highlighted by the participants is a need to unify the information material by only using the term “malignant melanoma” and not “skin cancer,” as this could contribute to a clarity of understanding melanoma.

#### 3.1.4. Awareness—To Know

The category “Awareness—to know,” described the participants’ need to prevent illness. The elderly men described different experiences of skin cancer, both benign and malignant, and experiences of skin cancer which can be manifested as birthmarks and freckles. Men described a lack of awareness that men over 65 years were at risk of a skin cancer such as malignant melanoma, which is why the booklet gave useful information that helped them to act on various skin changes. Furthermore, they described a feeling of alertness, accompanied by a form of curiosity to know, which was described as preparedness to act if skin changes arise. According to the participants, information should balance between clarity and a slight scariness to spark interest, because people need a kind of awareness. They argue that fear at a lower level could be helpful to keep alert and remain aware and focus on skin changes.

One aspect that has been highlighted by the informants is the threshold for men to make contact with health services for a health evaluation; this is experienced as lower when they already have contact with a physician. They described that the booklet gave them a boost to contact health services to check for skin cancer when they found it difficult and unsafe to do skin self-examination. Men made comparisons with the annual vehicle inspection that all cars must undergo, and stressed the need for the same consideration to the human body. Targeted health discussions dealing with lifestyle and health can lead to increased awareness and ability to prevent illness. 

According to the participants, caregivers could introduce a simple assessment option that includes skin examination, an “open house” or “quick cash” dealing with “easier” health problems, “drop-in” with shorter visit times that could be performed by a nurse instead of a physician. Informants argue that physicians can be used for more complicated cases of illness, which in turn may create better access to health services with shorter waiting time. Moreover, the men stressed that phone numbers and addresses to the nearest health center should be included in the booklet, to make it easier to choose the “right” contact route. Participants also found it time consuming and troublesome to call the health center, due to long waiting times and complicated instructions regarding different button commands, especially for an older audience. Participants described that the booklet is useful regarding the importance of information about preventive measures to reduce risk factors, which emphasized them to be aware of melanoma. One participant described it as:

*“... It was the personal approach where and then the folder that showed what it was all about, and images and some text, then I think most people read them, it should not be too much body text, but it has to be direct like that ... I do not just go and wait for it to happen, that I should suffer so to speak ...”*

The participants described the sun as both a strength, light or life and a threat, causing damage to skin cells. Awareness and knowledge of the sun’s harmful effects contribute to a higher degree of protection against strong sunlight. The men’s previous experience was of how they, in their early years, were exposed to the sun when it was common to go shirtless. Participants also pointed out changed travel patterns with longer trips abroad that contribute to stronger sun exposure. They have a greater awareness and knowledge about melanoma through healthcare (knowledge), industry (sunscreen products) and media attention (celebrities). A summary of the usefulness of the information booklet is awareness through knowledge that age is a natural explanation for skin changes.

### 3.2. Discussion

The aim of the study, to describe elderly men’s experience of information about malignant melanoma, was achieved. The conclusion of the current study can be described as interesting and useful information, which includes prevention and early detection. Informants were satisfied with the current information material and described that its contents added value and was perceived as reliable to improve security to act in terms of malignant melanoma. They pointed out that an effective distribution method was personal letters sent out by regular mail by a caregiver. However, it is also important to send out a reminder, for example as a postcard, inviting people to develop their knowledge about ill health and illness. The elderly men also emphasized that images were valuable as a supplement to a short text, helping them to understand and use the content, but also in discussions with others on melanoma.

The study shows the importance of how information should be presented with limited body text that is reinforced and exemplified by challenging and clear images in order to educate people about skin cancer and also how to do perform self-examinations. This is in line with other researchers’ findings [2,3,4,8,10]. The information can serve as support material to improve men’s participation in decisions on their various skin examinations, which is in line with the legislators’ intentions of good and effective healthcare [5,31,32]. This is one tool to develop a person-centered care, based on a holistic approach focusing on men’s need of security to act on specific risk factors through self-examination to identify melanoma at an early stage and therefore improve treatment options. A study by Grosch, Medvene and Wolcott [33] described that person-centered caregiving instruction based on a high level of interpersonal cognitive complexity, communication, and relationship-building skills, is one way to handle anxiety and other feelings when cancer is discussed. Moreover, men’s knowledge is changing due to their life experiences, which is a key point in person-centered care [34,35].

However, counseling healthcare services to men could lead to anxiety related to having cancer. To be able to improve elderly men’s health status, an information booklet could fill a gap of knowledge about malignant melanoma, in order to achieve a sense of coherence (SOC). Antonovsky’s [36] research about sense of coherence (SOC) and the theory of salutogenesis could be used focusing on health rather than illness. A healthy life including knowledge about sunbathing and skin changes could improve a sense of coherence for the population according to melanoma [32,36]. Our resources in healthcare is limited due to the fact that people live longer with higher demands on healthcare; it is therefore important to prevent or delay illness. It could be a significant tool to improve and use clients’ knowledge about illness as melanoma, as an untapped resource for the future. If health professionals could create empowering dialogs that enforce the strengths of elderly men and their relatives within the area of malignant melanoma, an educational process begins that includes a self-care situation consisting of comprehensibility, manageability and meaningfulness in life from a wellness factor.

By adopting person-centered care (PCC) as a care model may improve men’s experience of health services. However, PCC must be supported by the healthcare organization to use specific interventions that help caregivers to embrace person-centered care practices, which is in line with other researchers’ studies [33,37,38,39]. Educational resources produced using a person-centered care approach could provide health professionals with practical strategies on how to interact with elderly men and their need for health services and health information. Information material should encourage reflection and critical thinking about practical realities such as being elderly and male [40], which is in accordance with the participants’ need for action instead of waiting for illness. Informants also highlighted that self-care activities can be undertaken by any kind of extended control activities such as health conversation or other preventive actions once a year. For example, if a nurse works with active patient education and supports structure, it is possible to identify melanoma in an early stage and therefore improve treatment prognosis, which is supported by research in the field [8,10]. In order to provide educational guidance to do skin self-examination, a supplement about risk factors in the area of skin cancer is needed. Handy and Sankar [34] showed that men are willing and able to perform self-examination, but they need clear guidance and a variety of education tools to promote these health initiatives. According to this study, it is a challenge to educate elderly men about illness and the use of healthcare and social services due to a feeling of being an invisible population [41]. 

Research shows [42] that women use and rely more on cancer information and healthcare services than men, which is why it is a challenge for health professionals to increase elderly men’s trust in healthcare. A variety of media could be used as information channels, as a Loiselle and Dubois [42] study describes. Multimedia technology added value to cancer information, in the form of tailored informational support, and made it possible to inform about the utilization of healthcare services. Traditional formulations of masculinity may threaten men’s sense of control over their wellbeing. However, to improve knowledge, health professionals like nurses could be specifically trained in interpersonal communication skills to be able to meet elderly men’s information needs through SOC [21,36,43]. Vugt *et al*. [44] describe that providing information about the disease, sampling, and treatment, combined with risk estimation, enhances men’s decision making concerning their cancer disease. By being an informed decision maker, elderly men’s sense of coherence can be improved.

However, caregivers should increase their preventive work within the area of cancer (melanoma) by well-developed information booklets targeting different groups, such as elderly men. Davison and Breckon [45] stress that age has an impact on men’s role as healthcare consumers and argues that ongoing information needs to be addresses by a multi-disciplinary support program with access to both education sessions and written information. Moreover, information material specifically for men is rare, thereby suggesting it is important to develop available and clear information to create awareness and to be able to prevent illness or identify it in an early stage. Therefore, it is important to improve treatment options and prognosis within the area of melanoma, which is congruent with other researchers’ findings [46,47]. By taking care of men’s experiences of information material, men’s voices could be heard and used by health professionals to improve patient-centered healthcare service [48]. Moreover, it is important to develop a wider perspective on knowledge among men with malignant melanoma. Psychological support [8], for example by psychoeducational group, can decrease anxiety disorder and enhance effective coping [49]. Interventions might incorporate relative participation to solve misperceptions of support [50]. Another perspective is gender. Men seldom acknowledged interest in attention to bodily changes, but when awareness appears, they make a quick decision for an expert’s assessment and are more dismissing-avoidant in attachment [51,52]. Nordic countries—especially Swedish people—have high survival ratios (malignant melanoma), and one explanation could be national guidelines and public educational programs about skin examination in order to detect potential changes [53]. There are also results that highlight that social inequality in incidence rates (malignant melanoma) can be associated with higher social position [54]. In the Nordic countries, there are only minor differences between sexes, but mortality is higher among men [53], which is why improvement by patient-centeredness could ensure compliance with obligations in order to strengthen men’s position in health services. Health professionals should establish elderly men as partners in healthcare by engaging and involving them in health policies and decision making by being prepared using an information booklet about malignant melanoma. Moreover, to improve patient-centeredness, health professionals could sustain efforts to facilitate coordination and continuity of healthcare. However, to define a framework for assessment reflections and to strengthen healthcare patient-centeredness should be included using sense of coherence (SOC) and the theory of salutogenesis [32,36]. When clients feel secure about their health issues, health professionals could improve and allocate care and cure due to the population’s needs by using all available resources, including patients and their relatives’ self-care learning about melanoma due to skin self-examinations.

### 3.3. Limitations

The limitation of this study is the qualitative approach restricted to 15 interviews with elderly men. The selection process could be improved due to different experiences of melanoma, other diseases and healthcare that could influence the informants’ awareness about melanoma. The study’s validity could be challenged due to this limitation, and further studies are thus needed to develop the knowledge about elderly men’s experience of the information material about malignant melanoma in order to broaden perspectives. However, the trustworthiness of the results was ensured through a systematic scientific analysis using a well-documented methodology of manifested qualitative content analysis [29,55].

## 4. Conclusions

Security to act is an important perspective for caregivers to reflect on to be able to improve health information and health education for patients. In order to be aware of skin cancer, any information should also include information about risk factors to prevent melanoma. However, to improve knowledge, health professionals, such as nurses, could be specifically trained in interpersonal communication skills to be able to meet elderly men’s information needs through a sense of coherence. By using person-centered care, based on a holistic approach focusing on men’s need for security to act on specific risk factors and to do skin self-examination, illness could be identified early. However, availability of information material is one part; another part is clear information material so men couldunderstand its content. Finally, information should create awareness to do skin self-examination in order to prevent illness. Information material that is person-centered can improve elderly men’s experience of health and can contribute to a sense of coherence. Moreover, person-centered care focusing on elderly men’s experiences of getting on with life could provide support due to security through availability, as well as clarity and awareness to prevent illness.

The results of this study could help other caregivers to improve their information documents in the area of patient education to prevent illness. Health professionals’ perspectives of how information and health education should be developed is one part, the other part is to take people’s view about *Security—to act* on information material to prevent illness into account. However, *Availability—to use*, *Clarity—to understand*, and *Awareness—to know* were identified as factors that influenced men’s ability to engage in secondary prevention by raising awareness of signs and symptoms of early disease and how to perform skin self-examinations. Strategies identified in this article could help caregivers to develop and distribute educational information material to improve elderly men’s empowerment built on evidence-based knowledge from a person-centered care perspective.

## References

[1] Black L., Runken C., Eaddy M., Shah M. (2007). Chronic disease prevalence and burden in elderly men: An analysis of medical claims data. J. Health Care Finance.

[2] Cummins D., Cummins J., Pantle H., Silverman M., Leonard A., Chanmugam A. (2006). Cutaneous malignant melanoma. Mayo Clin. Proc..

[3] Markovic S.N., Erickson L.A., Rao R.D., McWilliams R.R., Kottschade L.A., Creagan E.T., Weenig R.H., Hand J.L., Pittelkow M.R., Pockaj B.A. (2007). Malignant melanoma in the 21st century, part 1: Epidemiology, risk factors, screening, prevention, and diagnosis. Mayo Clin. Proc..

[4] Knight C. (2011). Looking at skin cancer and effective sun protection. Br. J. Sch. Nurs..

[5] Swedish Association of Local Authorities and Regions, SALAR, National Board of Health and Welfare (2011). Öppna jämförelser av cancersjukvårdens kvalitet och effektivitet. Jämförelser mellan landsting 2011.

[6] Renzi C., Mastroeni S., Mannooranparampil M., Passarelli F., Potenza C., Pasquini P. (2011). Reliability of self-reported information on skin cancer among elderly patients with squamous cell carcinoma. AEP.

[7] Hodgetts J. (2011). Diagnosis and management of malignant melanoma. CancerNurs. Pract..

[8] Wheller T. (2006). Psychological consequences of malignant melanoma: Patients’ experiences and preferences. Nurs. Stand..

[9] Guile K., Nicholson S. (2004). Does knowledge influence melanoma-prone behavior? Awareness, exposure, and sun protection among five social groups. Oncol. Nurs. Forum.

[10] Wheller T. (2009). Nursing the patient with malignant melanoma: Early intervention. Br. J. Nurs..

[11] LaGasse N. (2011). Plastic surgery: Plastic surgeon and cosmetic dermatologist: Complications and liability. J. Leg. Nurs. Consult..

[12] Foster L. (2012). Raising men’s awareness of the need to monitor their health. Kai Tiaki Nurs. N. Z..

[13] Bungay H., Cappello R. (2009). “As long as the doctors know what they are doing”: Trust or ambivalence about patient information among elderly men with prostate cancer?. Eur. J. Cancer Care.

[14] Kelly D. (2009). Changed men: The embodied impact of prostate cancer. Qual. Health Res..

[15] Mendias E., Paar D. (2007). Perceptions of health and self-care learning needs of outpatients with HIV/AIDS. J. Commun. Health Nurs..

[16] Jackson G. (2007). Men health 2007: The need for action. Int. J. Clin. Pract..

[17] Nokes K., Nwakeze P. (2005). Assessing self-management information needs of persons living with HIV/AIDS. AIDS Patient Care STDS.

[18] Swedish Agency for Health and care Services & National Board of Health (2011). Öppna. Jämförelser av Cancersjukvårdens Kvalitet och Effektivitet. Jämförelser Mellan Landsting 2011.

[19] National Board of Health & Cancer fonden (2009). Populärvetenskapliga Fakta om Cancer Cancer. I Siffror 2009.

[20] Parks C., Turner M., Perry M., Lyons R., Chaney C., Hooper E., Conaway M.R., Burns S.M. (2011). Educational needs: What female patients want from their cardiovascular health care providers. Med. Surg. Nurs..

[21] Koutsopoulou S., Papathanassoglou E., Katapodi M., Patiraki E. (2010). A critical review of the evidence for nurses as information providers to cancer patients. J. Clin. Nurs..

[22] Shieh S.-C., Tung H.-S., Liang S.-Y. (2012). Social support as influencing primary family caregiver burden in taiwanese patients with colorectal cancer. J. Nurs. Scholarsh..

[23] Blows E., de Blas J., Scanion K., Richardson A., Ream E. (2011). Information and support for older women with breast cancer. Cancer Nurs. Pract..

[24] Rosengren K., Athlin E., Segesten K. (2007). Presence and availability: Staff conceptions of nursing leadership on an intensive care ward. J. Nurs. Manag..

[25] Rosengren K., Bondas T. (2010). Supporting “two-getherness”: Assumption for nurse managers working in a shared leadership model. Intens. Crit. Care Nurs..

[26] Gelberg J., McIvor A. (2010). Overcoming gaps in the management of chronic obstructive pulmonary disease in older patients. New insights. Drugs Aging.

[27] Quaglia A., Vercelli M., Lillini R., Mugno E., Coebergh J.W., Quinn M., Martinez-Garcia C., Capocaccia R., Micheli A. (2005). Socio-economic factors and health care system characteristics related to cancer survival in the elderly. A population-based analysis in 16 European countries (ELDCARE project). Crit. Rev. Oncol. Hematol..

[28] Polit D., Beck C.T. (2012). Nursing Research: Generating and Assessing Evidence for Nursing Practice.

[29] Egberg Thyme K., Wiberg B., Lundman B., Hällgren Graneheim U. (2013). Qualitative content analysis in art psychotherapy research: Concepts, procedures, and measures to reveal the latent meaning in pictures and the words attached to the pictures. Arts Psychother..

[30] Codex. Rules & Guidelines for Research. The Humanities and Social Sciences. http://www.codex.vr.se/en/forskninghumsam.shtml/.

[31] Swedish Code of Statutes. 2010:659 Patientsäkerhetslagen. (Patient Safety Act). http://www.socialstyrelsen.se/regelverk/lagarochforordningar/.

[32] Swedish Agency for Health and care Services Patient-Centeredness in Sweden’s Health System—An External Assessment and Six Steps for Progress. http://www.vardanalys.se/index.htm/.

[33] Grosch K., Medvene L., Wolcott H. (2008). Person-Centered caregiving instruction for geriatric nursing assistant students. J. Gerontol. Nurs..

[34] Handy P., Sankar N. (2008). Testicular self-examination—Knowledge of men attending a large Genito urinary medicine clinic. Health Educ. J..

[35] Ekman I., Wolf A., Olsson L.-E., Taft C., Dudas K., Schaufelberger M., Swedberg K. (2012). Effects of person-centered care in patients with chronic heart failure: The PCC-HF study. Eur. Heart J..

[36] Antonovsky A. (1987). Unravelling the Mystery of Health: How People Manage Stress and Stay Well.

[37] Crandall L., White D., Schuldheis S., Talerico K.A. (2007). Initiating person-centered care practices in long-term care facilities. J. Gerontol. Nurs..

[38] King S., O’Brien C., Edelman P., Fazio S. (2011). Evaluation of person-centered care essentials program: Importance of trainers in achieving targeted outcomes. Gerontol. Geriatr. Educ..

[39] Carlström E., Ekman I. (2012). Organisational culture and change: Implementing person-centered care. J. Health Organ. Manag..

[40] Bradley S.L., de Bellis A., Guerin P., Walters B., Wotherspoon A., Cecchin M., Paterson J. (2010). Reenacted case scenarios for undergraduate healthcare students to illustrate person-centered care in dementia. Educ. Gerontol..

[41] Kaye L., Crittenden J., Charland J. (2008). Invisible older men: What we know about older men’s use of healthcare and social services. Generations.

[42] Loiselle C., Dubios S. (2009). The impact of multimedia informational intervention on healthcare service use among women and men newly diagnosed with cancer. Cancer Nurs..

[43] Lindström B., Eriksson M. (2005). Salutogenesis. J. Epidemiol. Commun. Health.

[44] Vugt H.V., Roobol M., Venderbos L., Joosten-van Zwanenburg E., Essink-Bot M., Steyerberg E., Bangma C., Korfage I. (2010). Informed decision making on PSA testing for the detection of prostate cancer: An evaluation of a Leaflet with risk indicator. Eur. J. Cancer.

[45] Davison B., Breckon E. (2012). Factors influencing treatment decision making and information preferences of prostate cancer patients on active surveillance. Patient Educ. Couns..

[46] Brand S. (2010). Developing a service to support men with testicular cancer. Cancer Nurs. Pract..

[47] Townsend A. (2010). Support for men newly diagnosed with prostate cancer. Nurs. Stand..

[48] Mathers S., McKenzie G., Robertson E. (2011). A necessary evil: The experiences of men with prostate cancer undergoing imaging procedures. Radiography.

[49] Stark D., Kiely M., Smith A., Velikova G., House A., Selby P. (2002). Anxiety disorders in cancer patients: Their nature, associations, and relation to quality of life. J. Clin. Oncol..

[50] Lichtenthal W.G., Cruess D.G., Schuchter L.M., Ming M.E. (2003). Correspondence of recipient and provider perceptions of social support among patients diagnosed with or at risk for malignant melanoma. J. Health Psychol..

[51] Hajdarevic S., Schmitt-Egenolf M., Brulin C., Sundbom E., Hörnsten Å. (2011). Malignant melanoma: Gender patterns in care seeking for suspect marks. J. Clin. Nurs..

[52] Hamama-Raz Y. (2012). Does psychological adjustment of melanoma survivors differs between genders?. Psycho-Oncology.

[53] Tryggvadóttir L., Gislum M., Hakulinen T., Klint Å., Engholm G., Storm H.H., Bray F. (2010). Trends in the survival of patients diagnosed with malignant melanoma of the skin in the Nordic countries 1964ߝ2003 followed up to the end of 2006. Acta Oncol..

[54] Dalton S.O., Schüz J., Engholm G., Johansen C., Kjaer S.K., Steding-Jessen M., Storm H.H., Olsen J.H. (2008). Social inequality in incidence of and survival from cancer in a populationbased study in Denmark, 1994ߝ2003: Summary of findings. Eur. J. Cancer..

[55] Elo S., Kynga S.H. (2008). The qualitative content analysis process. J. Adv. Nurs..

